# The complete mitochondrial genome of *Phloeosinus perlatus* Chapuis, 1875 (Coleoptera: Scolytinae)

**DOI:** 10.1080/23802359.2021.1899082

**Published:** 2021-03-18

**Authors:** Xiaoqian Weng, Shaozhen Wang, Huan He, Mingqing Weng, Minghui Zhang, Songqing Wu

**Affiliations:** a College of Forestry, Fujian Agriculture and Forestry University, Fuzhou, China; b Key Laboratory of Integrated Pest Management in Ecological Forests, Fujian Province University, Fujian Agriculture and Forestry University, Fuzhou, China

**Keywords:** Complete mitochondrial genome, *Phloeosinus perlatus* Chapuis, phylogenetic analysis

## Abstract

*Phloeosinus perlatus* Chapuis, 1875 (Coleoptera: Scolytinae) is a major boring pest of Chinese firs. The length of the complete mitochondria genome of *P. perlatus* was 17,054 bp with 29.7% GC content, including 30.0% A, 11.3% C, 18.4% G and 40.3% T. The genome encoded 13 protein-coding genes, 22 tRNAs, and 2 rRNAs. Phylogenetic analysis showed that *P. perlatus* was closely related to *Scolytus seulensis*. This study provided useful genetic information for the subsequent studying the prevention of *P. perlatus*.

*Phloeosinus perlatus* Chapuis is a major boring pest of Chinese firs, because the adults and larvae tunnel beneath the bark, such tunneling can accelerate plant wilt and death (Zhao and Cao 1987). This pest mainly distributed in partial Asian countries (China, Japan, Korea), while in China it presented in Anhui, Zhejiang, Jiangxi, Fujian, Henan, Shandong, Sichuan and Taiwan Provinces, etc. One generation was produced annually with the adults overwintering (Zhao and Cao 1987). However, no reports about the genetic evolution analysis of *P. perlatus* have been published until now. In this study, we reported the complete mitochondrial genome of *P. perlatus* based on Illumina sequencing data and investigated the phylogenetic relationship by maximum-likelihood tree inference method. The results could provide an important genetic information to study the genetic evolution of *P. perlatus*.

The *P. perlatus* adults were collected from Minhou county, Fujian province, China (119.2223E, 25.9068 N) by the traps with sexual attractants. The specimens were deposited at the Fujian Agriculture and Forestry University (URL: https://lxy.fafu.edu.cn, contact person: Jiayi Ma and email: 790167087@qq.com) under the voucher number TN-202101. Total genomic DNA were extracted from several individuals using TruSeq DNA sample Preparation Kit (Vazyme, China), and purified by QIAquick Gel Extraction Kit (Qiagen, GER). DNA quality and concentration were determined using Nanodrop (Thermo Fisher Scientific, USA). DNA sequencing was performed by Illumina Hiseq 2500 (Illumina, USA). A total of 53,195,560 clean reads were obtained from the 57,498,476 raw reads after filtration. The clean reads were assembled by using MitoZ and metaSPAdes (Nurk et al. 2017). Then the assembly sequence was annotated by the MITOs web server (Matthias et al. 2013). And tRNA genes were predicted using tRNAscan software (Lowe and Eddy 1997). The complete mitochondrial genome sequence of *P. perlatus* has been submitted to NCBI GenBank with accession number MW447510. The complete mitochondria genome of *P. perlatus* forms a circular structure covering 17,054 bp in length, with 13 protein-coding genes, 22 tRNAs, and 2 rRNAs. The GC content of the complete genome was 29.7%, including 30.0% A, 11.3% C, 18.4% G and 40.3% T.

In order to confirm the phylogenetic position of *P. perlatus*, the evolutionary tree was constructed with related 17 different species insects by MEGAX using Maximum Likelihood tree model with 1000 bootstrap replicates (Hall 2013). The phylogenetic tree showed that the *P. perlatus* was in close association with *Scolytus seulensis* (Coleoptera: Scolytinae) (Figure 1). The complete mitochondrial genome of *P. perlatus* will provide useful genetic information for increasing the richness of the Scolytinae, as well as assisting in phylogenetic and evolutionary studies of Scolytinae.

**Figure 1.**
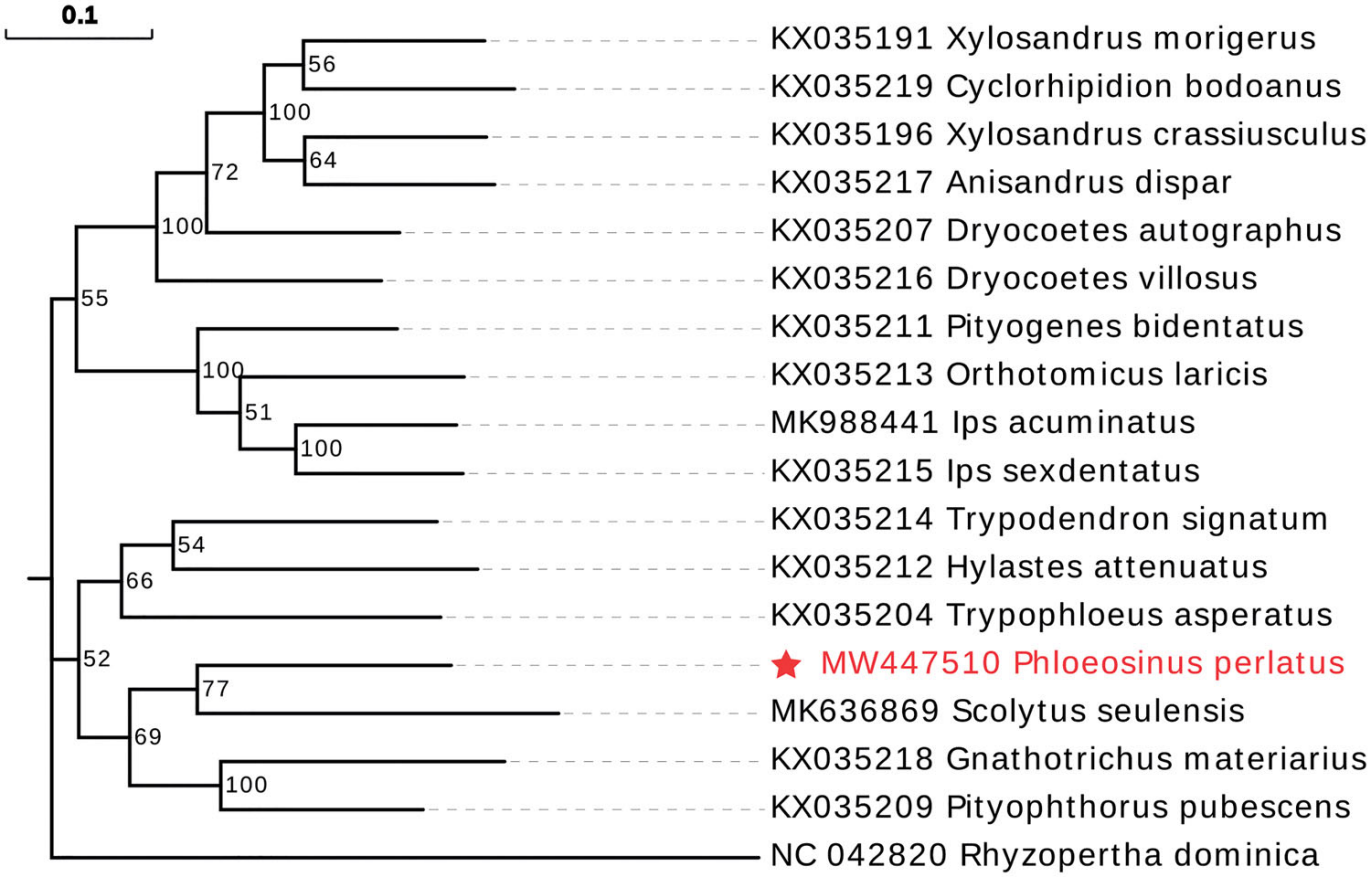
Maximum-likelihood tree of *Phloeosinus perlatus* Chapuis and related 17 different species insects based on the mitochondrial genome. Bootstrap support values are labeled near the branch.

## Data Availability

The genome sequence data that support the findings of this study are openly available in GenBank of NCBI at https://www.ncbi.nlm.nih.gov under the assession no. MW447510. The associated BioProject, SRA and Bio-Sample number was PRJNA689703, SRX9785739, and SAMN17214850 respectively.
